# An ecological systems model of employee experience in industry-led autism employment programmes

**DOI:** 10.1177/13623613241241574

**Published:** 2024-03-29

**Authors:** Simon M Bury, Rosslynn Zulla, Jennifer R Spoor, Rebecca L Flower, David B Nicholas, Darren Hedley

**Affiliations:** 1 La Trobe University, Australia; 2 University of Calgary, Canada; 3 University of Alberta, Canada

**Keywords:** autism employment, autistic adults, ecological systems, supported employment

## Abstract

Industry-led employment programmes have emerged to transition autistic people into employment and meet industry labour needs. However, theoretical research is limited in this area, often failing to appreciate the influence of the broader employment ecosystem. In this study, we interviewed 33 autistic employees (*n* = 29 males, *M*_age_ = 29.00 years) from two industry-led employment programmes regarding their experience of the programme’s supports, relationships and impact. We used qualitative content analysis to identify five themes: (1) working involves multiple job tasks that evolve as the employment context changes; (2) workplace relations are diverse and shaped by the type of work and the work environment; (3) workplace needs evolve as autistic individuals navigate the work environment; (4) developing a professional identity in the workplace through mastery and integration; and (5) recommendations for the development of supportive workplace environments for autistic individuals. We describe the way that factors within (e.g. training) and outside the two employment programmes changed and interacted over time to contribute to the participant’s work experience and professional identity. Building on ecological systems theory, our unique contribution to the literature is a new model capturing individual and workplace factors that contribute to the work experience of autistic people who participate in industry employment programmes.

**Lay Abstract:**

We asked 33 autistic adults from two industry-led employment programmes about their experiences in the programmes. These are programmes started by companies to recruit and support autistic people in work. We also asked about their workplace supports, relationships and how they thought the programme had impacted their life. Understanding the experiences of people in these industry-led employment programmes is important as the information can help to improve the programmes and participants’ experiences. After reviewing the interviews, we found five themes that best described the employee’s experience: (1) working involves multiple job tasks that evolve as the employment context changes; (2) relationships in the workplace are diverse and are influenced by the type of work participants do and the work environment; (3) workplace needs change as the autistic employees learn to navigate their work environment; (4) autistic employees develop a professional identity in the workplace as they master work and feel more integrated in the workplace; and (5) recommendations for the development of supportive workplace environments for autistic people. We explored the way that aspects of the two employment programmes (e.g. training) and factors outside the programme changed with time and contributed to the participant’s experience. We developed a new model to capture individual and workplace factors that contribute to the experience of autistic people who participate in industry employment programmes.

Autistic people^1^ often report a strong desire to work (Chen et al., 2015) and develop financial independence (Hedley, Cai, et al., 2018), yet experience significant challenges in finding long-term and meaningful employment (Nicholas et al., 2019; van Heijst & Geurts, 2015). In Western countries where data are available, this is reflected in low rates of employment (e.g. 27.3% in Australia, Australian Bureau of Statistics, 2019; 14% in the United States & Canada, Roux et al., 2017; Zwicker et al., 2017) compared to non-autistic populations and high rates of underemployment or working in roles that do not match skills or training (Hedley et al., 2023).

There is some evidence that autistic traits and behaviours, such as differences in social communication (Bury, Flower, et al., 2021; Nicholas & Klag, 2020), repetitive and restrictive behaviours (Bury, Hedley, & Uljarević, 2021; Chen et al., 2015), and sensory differences (Pfeiffer et al., 2017), contribute to the barriers autistic people face when seeking employment, reflecting the fact that most work environments and organisational processes fail to meet the needs of autistic employees. Moreover, autistic job seekers report challenges navigating the job search process (Richards et al., 2019), engaging in the job interview (a traditionally social process; Finn et al., 2023), and uncertainty as to whether it is safe to disclose their autistic identity (Finn et al., 2023). This latter point may be justified, as disclosure of an autistic identity has been found to impact employer perceptions of a simulated autistic employee (Flower et al., 2021). However, autistic people who are provided with a supportive work environment, including physical or sensory accommodations, supportive colleagues and job roles that are well-matched to their interests and skills, are often reliable and high-performing (Hedley et al., 2023). Indeed, Hedley et al. (2023) found that, when provided with appropriate supports (as part of an industry-led supported employment programme), a majority of unemployed autistic people were able to transition to stable, open employment that was matched to their skills and qualifications. It is probable that the challenges many autistic people experience obtaining and sustaining employment reflect a poor person–environment fit, lack of accommodations or lack of autism understanding, rather than a lack of skills or ability to function in mainstream work environments.

Industry-led autism employment programmes (Austin & Pisano, 2017; Hedley et al., 2023) use modified human resource management (HRM) processes and supportive work environments to address the barriers to both obtaining and maintaining employment matched to skills and education. However, despite increasing numbers of programmes, there is limited research available describing these programmes, the effectiveness of their processes or the experience of those involved (Spoor et al., 2024). From the available research, these programmes can provide significant benefits in terms of immediate employment success (Flower et al., 2019; Hedley, Spoor, et al., 2018) and competitive employment success post-programme completion (Hedley et al., 2023), as well as financial benefits to autistic employees (e.g. wages) and government (e.g. welfare savings; Hedley et al., 2023).

Autistic employees within these programmes report an ‘anxious optimism’ about starting in the programmes (Remington & Pellicano, 2019), with qualitative research supporting better well-being outcomes, including, life balance, self-esteem and sense of purpose (Flower et al., 2019; Hedley, Cai, et al., 2018). However, well-being is unchanged in longitudinal quantitative studies (Hedley et al., 2019; Remington et al., 2022). Facilitators of success perceived by autistic employees include strong support network outside of work (Remington et al., 2022), targeted and appropriate supports (Flower et al., 2019; Remington & Pellicano, 2019), with adjusting to aspects of the role (Hedley, Cai, et al., 2018), and communication challenges (Remington & Pellicano, 2019) as potential barriers. Co-workers report individual (e.g. autism knowledge) and organisational benefits (e.g. organisational reputation, organisational pride; Grenawalt et al., 2020; Spoor, Bury, et al., 2021) to such programmes, but they have also reported challenges associated with perceived workload concerns and their engagement with these programmes (Spoor, Flower et al., 2021), and concerns regarding integration beyond the programme (Spoor, Bury, et al., 2021), but not in all studies (Grenawalt et al., 2020). While these studies report facilitators and barriers at the individual or co-worker level, very little research has considered the broader organisation at the ecosystem level within autism employment (Vogus & Taylor, 2018).

While individual approaches are important to understanding challenges autistic people may encounter in the workplace (Bury et al., 2019, 2020), employment outcomes and experiences occur within a broader ecosystem (Nicholas et al., 2018). Nicholas et al. note that while autistic people bring a personal set of individual traits and assets (e.g. cognitive ability, prior job experience, work ethic), system-level factors (e.g. workplace relationships, supervisors, physical work environment, policy) also have a substantial impact on employee experience and outcomes. In the context of industry-led autism employment programmes, this means it is essential to understand how the interplay of the employment programme itself with workplace factors (e.g. social relationships, support) serves as barriers and facilitators to positive workplace experiences for autistic individuals.

## Research context: supported employment programmes for autistic people

The autism employment initiatives described in this study refer to two similar, industry-led supported employment programmes that aim to provide autistic people with a competitive role and career path within the public or private sector. As both programmes are open only to autistic candidates, provide competitive pay, but with autism-specific supports, we believe the term ‘supported employment’ best describes the programmes, although we acknowledge that the term is often used to describe non-competitive employment elsewhere. These programmes have been described elsewhere (Flower et al., 2019; Hedley et al., 2023; Spoor, Bury, et al., 2021), although we provide a general overview here for the reader. In Programme 1, autistic staff were employed in 3-year contract roles by a large information and communications technology (ICT) services firm and worked in client organisation sites in federal government departments (Hedley et al., 2019, 2023; Spoor, Bury, et al., 2021). In Programme 2, autistic staff were employed by a large state government department for 1-year roles in records management, and the programme serves as a potential pathway into a career in the public service (Flower et al., 2019). In both programmes, autistic staff were employed in graduate entry-level roles and received salaries equivalent to peers in similar roles. Existing employees at each site received communications about the programme and were offered optional autism training. Modified HRM practices included selection based on an extended period of paid assessment and training. Successful candidates worked in autistic teams of eight to 14 people and had access to internal and external autism-specific support for at least the first 3 months.

## Current study

Given that research to date has failed to fully appreciate the potential for various elements both internal and external to the employee to shape their employment experience, this study will address this gap by considering how broader ecosystem elements impact employee experience. Specifically, in a series of qualitative interviews, we asked autistic employees engaged in two industry-led employment programmes how participants’ experiences of workplace support, relationships and programme elements impact their experience and success within the workplace with an intention of better understanding both the internal and external elements that might impact on their experiences and success. In this study, our goal was to develop a new model reflecting the interactive nature of individual- and system-level factors underpinning employment outcomes and identity for autistic employees. Our intention was that the model could be used to improve practice and policies, thereby leading to improved employment outcomes for this group.

## Methods

### Participants

Participants comprised 33 autistic employees (*M*_age_ = 29.00, *SD* = 8.84; *n* *=* 4 females, *n* *=* 29 males), comprising 80% of participants in Programme 1 and 71% of participants in Programme 2. Around half (54.5%) of participants were employed part-time (e.g. 28–38 h per week), with the rest employed full-time. Most had undertaken secondary education (33.3%) or post-secondary vocational training (e.g., Technical and Further Education, TAFE; 30.3%), with fewer with a graduate (24.2%) or postgraduate degree (6.1%; missing = 6.1%). Both programmes required evidence of a formal autism diagnosis.

### Procedure

Ethics approval was obtained by the La Trobe University Human Research Ethics Committee.

Programme 1 included three sites. Depending on the site they were employed at, participants were interviewed at 10, 18 or 30 months after programme commencement. Participants in Programme 2 were interviewed 4 months following commencement. Interviews were approximately 40 min and held at the organisation in a private meeting room. Participants were provided options of virtual (i.e. Zoom, telephone) or off-site interviews, but all elected to complete them onsite.

Interviews were semi-structured and were centred around the participants’ experience of workplace support (e.g. ‘How supported do you feel in your work?’), relationships (‘Tell me about your relationship with your co-workers (who are not in the (programme)?’), sharing of diagnosis (‘As part of the employment programme, your autism diagnosis has been disclosed to your employer and your co-workers. Has this disclosure impacted you?’), experience of the programme (‘Thinking back to when you started the programme, can you discuss whether your expectations you originally had about the programme have been met?’) and impact (‘Has this programme affected your life outside of work? Can you explain how?’).

### Data analysis

Following verbatim transcription, interviews were subjected to qualitative content analysis (Elo & Kyngäs, 2008; Graneheim & Lundman, 2004). NVivo qualitative data analysis and management software was used to conduct this analysis by R.Z., with a portion of the data also reviewed by team members (S.M.B. and D.B.N.). Content analysis is a useful approach for exploratory examination of phenomena that is not yet well-known or conceptualised (Liamputtong, 2020). Transcripts were analysed using a three-step process: (1) reading transcripts line-by-line (*preparation*), (2) conducting an inductive analysis through seeking meaning units (*organising*) and (3) generating a map that organised meaning units (*reporting*). This inductive approach involved coding meaning units (open coding), organising these units (categorisation) and interpreting underlying meanings of these units (theme generation). Rigour was ensured by reviewing codes, categories and themes with research team members with experience in qualitative data analysis and expertise in autism and employment to ensure resonance and ‘fit’ of findings (Elo & Kyngäs, 2008; Graneheim & Lundman, 2004; Guba & Lincoln, 2005). Referential adequacy ensured sufficient confirmatory data text quotes to allow confirmation of identified themes (Guba, 1981). The research team comprised non-autistic individuals, collectively with decades of experience in employment support in autism in Australia and internationally as well as experience in qualitative methodology.

### Community involvement

While the aims of this study were in line with research priorities of the Australian autistic community (Australian Bureau of Statistics, 2019), and the team included neurodivergent (though non-autistic) researchers, autistic people were not involved in the design, data collection, analysis or interpretation of this research.

## Results

Participant responses reflected on interactional elements in their experience and work environment, with five key themes emerging with three to four subthemes: (1) working involves multiple job tasks that evolve as the employment context changes; (2) workplace relations are diverse and shaped by the type of work and the work environment; (3) workplace needs evolve as autistic individuals navigate the work environment; (4) developing a professional identity in the workplace through mastery and integration; and (5) recommendations for the development of supportive workplace environments for autistic people. These themes are described below, including corroborative interview quotes.

### Theme 1. Working involves multiple job tasks that evolve as the employment context changes

Theme 1 speaks to participants’ experience of job tasks, and how their engagement and experience with work tasks change over time due to external and internal influences (see Table 1 for additional quotes for each subtheme).

**Table 1.** Themes, subthemes and quotes for Theme 1.

**Theme 1: working involves multiple job tasks that evolve as the employment context changes**				
Subthemes	Job tasks engaged in the workplace	How job tasks were experienced	Work environment influences work productivity	External and internal influences on work experience
	I often check – we test various services for our clients which is making sure that all their services are in peak condition and high quality before it’s released to the customer. When it comes to testing, we need to make sure we test again, and again, to make sure I say that everything is working properly. (Participant 13)	. . . my workload is pretty much all over the place as one month I could get a lot of tasks that are due in a couple of months. So that would leave me rather busy for a couple of months, but then on one week I could get multiple tasks too by the end of that, so it’s all over the place. (Participant 4)	The only thing I could think of is literally, the room we work in is a bit stuffy . . . therefore because the room we’re in, people can [get] sick very fast and because I’m always the – I’m the canary in the mine kind of thing. I’m the one that gets sick instantly. I get sick more often but that has nothing – no one could’ve done anything and my manager has raised it a million times. (Participant 27)	By car. I’m too far out. No public transport that would get me here. I get up at seven in the morning to get here at nine. I don’t get a lot of time in the mornings. (Participant 1)
**Theme 2: workplace relations are diverse and shaped by the type of work and the work environment**				
Subthemes	Relationships are dynamic and contingent on job task and/or environment	Relationships with autistic co-workers	Relationships with non-autistic co-workers	
	There is a little bit of a quirk when communicating with people outside of the POD because we’re actually physically separated, so that’s created a little bit of distance – that we’ve had trouble engaging with people. That’s improved a lot over the last couple of months. (Participant 31)	Yeah, it’s been really good. It’s been easy to talk with some of these people. Like they were all aware that everyone else is socially awkward and not expecting much from each other, socially. So that made it easier to make friends. There have been people – I’ve gone to their houses pretty often and stuff like that. Then everyone else – we’re comfortable approaching each other with everything. (Participant 12)	I have over the time very slowly started talking to people, and I enjoy those interactions. I don’t think I’d be able to do more over time, most of my job involved sitting in front of a computer, entering data in hours upon end, it doesn’t lead to a lot of interaction. But when you go to the printer or whatever, oh, how’s it going? What are you up to today? What did you get up to over the weekend? (Participant 23)	
**Theme 3: workplace needs evolve as autistic individuals navigate the work environment**				
Subthemes	Support varies as individuals adapts to their workplace	Types of support	Influences of organisational factors	Influence of training and support resources
	So, initially there was a lot of [autism specialist] consultation, I had a lot of support from the tech leads. That has tapered off a little bit. With managing Uni, that’s changed, depending on my workload. (Participant 30)	I get help with the technical content. If I want help with the explanations or anything, I’ll – I write out something that sounds good to me and then I go to our [Programme Organisation] technical supervisor kind of thing, whatever the relationship is there. I forget and just say hey, does this look good? (Participant 1)	So when we started it was very supportive – well it still is very supportive now but when we started it was pretty well supported and we’ve had a pretty weird fluctuation of support staff come through. We had one guy who was here for a few weeks and then left because of family issues. We’ve had a lot of support at one time, maybe I think it was five members in support. Now we’re down to two just because of how the company kind of operates at the moment. (Participant 13)	I think the programme has and also based on my other experience as well. But also, I think the programme – I think what helped us get used to the job was the fact that we got challenges, when we were doing the [Programme Organisation] training and the challenges were really useful because it helped us know what we were getting prepared for. (Participant 25)
**Theme 4: developing a professional identity in the workplace through mastery and integration**				
Subthemes	Work and autism identity	Engagement and mastery	Felt sense of integration	Relational and environmental factors influencing integration
	So I was able to give them my best, and well, I have no intent of letting them down because you get bosses like that, and you don’t want to let them down. So I give my best. Not any more than my best because that’s the best I can do. (Participant 23)	Yeah. I actually like having a lot of work, so I tend to make a lot of lists and schedule myself and figure out, I need to get this done by then. If I can’t do that, I either talk to the manager saying, look, this isn’t going to work timewise. Or I ask other people for help. Or the manager might go, this person’s free. Grab them on board. It’s really just communicating about it and making sure everyone’s on the same page. (Participant 28)	I definitely feel part of the broader team, especially the service ops branch, but not the rest of the department though. It is a pretty big department. (Participant 3)	I think a lot of it sort of, it feels, you feel a little bit separated, I think it’s basically because of the physical separation in the building. Because the office where they’ve put us in, is further apart from everywhere else. But like everybody, myself and everybody that’s actually taken, sit next to everybody else jobs, yeah I think it feels a lot more part of the team type of thing. (Participant 33)
**Theme 5: recommendations for the development of supportive workplace environments for autistic individuals**				
Subthemes	Need for adaptive resources/support	Need for feedback	Need for team-oriented support	
	So, sometimes it’s hard to recognise changes when you’re living through them. But as we’ve been here for more time, and we get more used to the task, they probably expect a slightly higher standard out of us. But the training was really good and it prepared us well for it. (Participant 24)	I think there could be more understanding that some people don’t – there could be more understanding that some people don’t think straight away certain things socially. That maybe backing off a bit and understanding why someone’s thinking that, and then correcting it. Rather than just saying, you’re not supposed to do this, you’re not supposed to do that. (Participant 6)	Well at the start the support was a bit more hands on in order to get to grips with the system and I needed as much assistance as possible from test leads, test managers and even people that – and STAs. As it went on I needed them less and plus I used other people from outside the [Programme Organisation] team to provide the necessary assistance that I needed. (Participant 14)	

#### Job tasks engaged in the workplace

Most participants described employment to largely involve multiple job tasks that required ongoing coordination and personal effort to meet workplace demands. Job tasks varied and were contingent on the department/work ethos in which individuals worked. Tasks completed included integrating protocols, documenting processes, creating and monitoring automation processes, monitoring and addressing ICT security issues, developing software applications to increase ICT security, analysing data, assisting in testing computer software and systems and archiving (e.g. digitising physical documents into a computer database).

#### How job tasks were experienced

Half of the participants enjoyed their work tasks, but some viewed these as too easy or repetitive, as recounted by one participant:

A lot of the time there is a lot of repetition with the work, it can get very dull with that. Having moved onto the programming, some of that has been rather unfamiliar and a bit uncertain because of that. It hasn’t really been feeling like anything has been too hard. Possibly it could have been harder without getting to be a problem, but I wouldn’t really call it too easy either. (Participant 28)

The type, duration and volume of work varied depending on job task, the co-workers and the client/customer’s needs, where applicable. Participants felt work assignments often involved navigating between (1) independent and co-operative tasks, and (2) periods of slow activity to sudden intense work demand. These shifts generally were manageable for some participants due to the support of their managers as a participant highlighted:

For the most part, [supervisors] they made sure . . . to give us a workload based on – like we just do as much as we can. And . . . they gave us feedback to help us improve our work over time. But the workload was never too much because they only just got us to do what we could do which, once you get good at the job, means you’ll be doing more work. Sometimes they requested overtime from us. They never forced it on us. We had that opportunity there to work and get stuff done that way. (Participant 12)

Several participants learned to adapt, particularly during slow periods, by engaging in other tasks (e.g. reading, completing training exercises) and becoming self-reliant in doing tasks and the workflow. This included learning to manage tasks and deadlines (e.g. creating a work plan), adapting to challenges within (e.g. learning to work within teams towards deadlines) and outside (e.g. learning to complete tasks even when computer systems are down) their control and coping with personal and physical exertion and strain (e.g. learning to manage fatigue). These areas imposed varying levels of difficulty, which was sometimes mediated by support offered in the workplace.

#### Work environment influences work productivity

While work programmes were designed to accommodate sensory needs of the autistic employees, some participants still reported challenges with the sensory environment. For instance, some individuals stated that workspaces were noisy, had overly bright lights, felt too cool or warm, had off-putting smells and felt congested: factors that hindered their productivity and sense of well-being. Some autistic individuals adapted their workspaces through processes or resources such as headphones. Others requested adjustments such as a workspace with less noise/congestion. For a few individuals, accommodations were still being reviewed by senior administration at the time of participation.

#### External and internal influences on work experience

Workplace productivity was influenced by factors external to the workplace. Most participants spoke of learning to manage daily life, study responsibilities or interpersonal relationships while trying to maintain their productivity at work. Some independently learned to adapt (e.g. gaining sleep by going to bed earlier) while others received support from their managers (e.g. flexible work schedule). Work productivity was also influenced by individual and environmental assets. For instance, prior education (e.g. having a degree) or past work experience, in some cases, helped the individual navigate work tasks and manage workflows. Internal (e.g. autistic co-workers) and external (e.g. a counsellor) support was described to help balance workplace demands.

### Theme 2. Workplace relations are diverse and shaped by the type of work and the work environment

Theme 2 describes the nature of workplace relationships, and how the nature of the work and work environment can influence these relationships. The first subtheme provides a broad description of this theme, with two additional subthemes describing in the context of relationship with autistic or non-autistic colleagues.

#### Relationships are dynamic and contingent on job task and environment

Workplace relationships were described as *not* fixed as participants often worked with a wide range of autistic and non-autistic co-workers, managers and support workers. However, autistic individuals described often remaining together as a supportive cohort. They conveyed multiple social tasks and relational/interactional demands, including navigating video calls, managing professional and spam emails, respecting organisational hierarchy (e.g. requesting approval for a task from a manager), having informal conversations within (e.g. remembering names) and outside the workplace (e.g. attending social gatherings) and requesting feedback from co-workers. The nature of social relationships changed as the autistic employees experienced different types of social interactions. Conversations with autistic and non-autistic co-workers were generally characterised as positive but differed in nature, purpose and focus.

#### Relationships with autistic co-workers

Most participants described getting along well with their autistic peers, that is, having conversation, feeling close and ‘hanging out’ within (e.g. having lunch together) and outside the workplace (e.g. going to each others’ homes):

I would say I’ve been actually amazed at how close-knit our group has become . . . I had New Year’s celebration at my place and . . . six [colleagues] were there. We’ve done escape rooms and dinners and just general stuff because we’ve actually become very close and . . . that’s amazing because you don’t always have chances like that. (Participant 27)

Many participants perceived autistic peer co-workers as sources of support (e.g. learning/teaching how to do work tasks) and described broadening their understanding of how to work with other autistic individuals. For a few participants, their strong relationship with autistic peer co-workers helped them deal with life transitions (e.g. how to live independently).

#### Relationships with non-autistic co-workers

Conversations with non-autistic co-workers were described as largely informal and work-related. A few participants reported deepening their relationship with non-autistic co-workers as they shared personal stories in the workplace and attended social gatherings outside the workplace.

Sharing one’s autism diagnosis was required for involvement in the two programmes, for some this was seen as a positive means to employment and support and many participants did not feel they were treated differently because of divulging their diagnosis. Some felt that disclosure, along with awareness training, developed heightened awareness about autism that facilitated relationships with non-autistic co-workers:

I feel a lot more relaxed around [non–autistic peers] now. At first, I was a bit anxious about being the new kid on the block . . . the awareness sessions and getting to know them more and disclose . . . seems to have changed pretty much for the better – our relationship with them. Not that it was bad before; it’s just that I feel more comfortable around them now. (Participant 24)

### Theme 3. Workplace needs evolve as autistic individuals navigate the work environment

Theme 3 describes the evolution and nature of support needs, and the impact that organisational factors and autism knowledge have on support provided.

#### Support varies as individuals adapt to their workplace

Workplace support needs evolved and shifted as participants navigated the work environment. Received support often occurred earlier in employment, particularly as participants learned how to complete work tasks and engage with their co-workers and managers. As participants progressed in the workplace over time, support needs were less frequent, and when needed and sought/received, support generally was tailored to the individual’s specific needs. When participants moved to different areas of work within their department, support was often provided, particularly at the beginning.

#### Types of support

Support needs of autistic individuals varied, but included technical (e.g. learning to write an effective report), social (e.g. learning in-person and online social etiquette and requesting feedback), navigation (e.g. learning and adapting to new workplace protocols) and management/executive functioning (e.g. learning to manage stressful days) needs. Most autistic individuals generally felt supported in their workplace by their autism support worker (in one of the two programmes), a technical staff member and/or their manager. A few received external supports (e.g. a mentor, a counsellor) that were obtained prior to or during their employment. Participants characterised positive support as engagement with someone who was affirming and helpful; had autism knowledge; was encouraging, available, accommodating and understanding; and helped the individual adapt to their work environment. In contrast, inadequate or insufficient support was described as another’s lack of understanding about their needs in the workplace, and a lack of respect. While autism-focused support was valued by some, several felt that they did not specifically require autism-focused workplace support.

#### Influences of organisational factors

Organisational factors were described to have shaped how support was provided to, and experienced by, participants. For instance, autism support workers in the multi-site programmes were often transient and provided support to a large number of autistic employees, thus limiting their availability to focus on the individual. They also recounted how autism support workers and managers deliberated on who should be responsible for providing support to employees and how much work should be given to each employee. These deliberations, often occurring somewhat external or peripheral to the employee, reportedly had a bearing on the support received and how it was experienced by the individual.

#### Influence of training and support resources

Workplace support was also shaped by training and other resources offered to autistic employees and their non-autistic co-workers. Most participants felt that autism awareness training for non-autistic workers was helpful in broadening autism knowledge; however, several found these training sessions sometimes focused too heavily on clinical features or stereotypic presentations of autism. Mobile applications were also used in one of the two programmes to provide remote and individualised support to autistic employees, but scaling this tailored approach to all employees became difficult, as recalled by an individual:

The app’s a perfect example [of what was viewed as misguided scaling of an individualized approach the organisation took] an individualised approach which works, but then they want to mass produce it and then put it in an app form because it’s a cost-saving measure. It didn’t work. I wouldn’t have minded it if it was used as a supporting tool in addition to [other support], but they wanted it to be purely [as follows:] this is the app you use to support yourself in the workplace. So pretty much replacing [internal support workers] with an app was just a horrible idea. (Participant 31)

### Theme 4. Developing a professional identity in the workplace through mastery and integration

Participants described how their experience within the work environment shaped their professional identity within the workplace.

#### Work and autism identity

Participants’ experiences in the workplace were viewed to shape their identity as an employee and how they viewed themselves in that role. They described multiple traits that were reflective of an effective employee, including being attentive, flexible, self-motivated, and punctual, all in the aim of being productive.

For some, the disclosure of their autism diagnosis led to introspection and adjustment of their autism identity in the workplace. For others, disclosure challenged their sense of identity either due to a novel public disclosure of a diagnosis not shared with friends or grappling with categorisation under a different diagnostic label (e.g. Asperger’s vs autism). Others felt that disclosure linked their effectiveness as an employee with their autism identity in a positive way:

[The organisation] has actually found that [autistic] people [like] us . . . tend to have a higher output than generally people without it . . . having that disclosure has actually made them think, ‘oh, these people are going to be more valuable employees’. (Participant 14)

#### Engagement and mastery

Learning how to effectively engage in the workplace emerged from observing workplace colleagues (e.g. autistic and non-autistic co-workers, managers and support workers), being motivated to do well in the workplace and adapting over time to the nature and demands of the work and organisation. While many participants described the workplace experience as initially challenging, this experience generally improved as they learned how to navigate multiple tasks (e.g. learning to complete tasks faster, designing and implementing a work plan, learning to request support to complete a task). As participants gained mastery in their work environment and tasks through engaging in a supportive environment in which they could incrementally experience increased mastery, they viewed improvement in their sense of employment self-efficacy:

I could move into the work I’m already doing with other people now and not have a worry because I feel like I can do it independently. To begin with, I had a lot less – what do you call it – good feeling about myself . . . I had less confidence in my abilities, [and in] myself as a person, so I was scared to bring stuff up. But now I’m like, just ask. (Participant 27)

#### Felt sense of integration

Participants’ level of engagement and socialisation helped gain a sense of how they were perceived in the workplace as an employee. To be validated at work was largely based on participants’ perceptions of feeling integrated in the workplace. They described integration as reflective of a part of collective activities such as being included in team meetings, completing work as a team, having informal chats with co-workers and receiving support from co-workers and managers. A sense of integration reportedly was also shaped by the degree to which they related to others. It was further described as a social developmental process of learning how to be part of a team as they completed their work:

Taking like a month to go through training as part of [the] onboarding process . . ., [I] end up feeling quite integrated because . . . slowly as you keep coming in, you just start talking to that person and . . . you start feeling more integrated. Now, I feel quite comfortable and integrated . . . I kind of know [where] I am with respect to the department. (Participant 22)

#### Relational and environmental factors influencing integration

Relational and environmental factors played a role in shaping perceptions of integration. For instance, several participants described how work integration was facilitated by common traits (e.g. education, age) and relationships with other autistic co-workers. Physical workspaces further shaped participants’ sense of integration, as negatively exemplified by participants who attributed not feeling a part of their team to external (distant) job placements. Conversely, other participants attributed integration to co-operative work with colleagues, a supportive work culture, structural elements (e.g. work processes organised in ways that engaged the individual in a supportive way), and work/people stability. Reflecting on elements the contribute to and detract from integration, a participant commented,

When we’re working on projects we’re quite often working with people from other sections, that can be quite helpful so that’s integrated. But then we’re also contractors so that does set us apart from the public service culture. Because the public service culture [has] a strong collective identity and they foster that identity, so being a contractor does mean that we’re not quite one of the family, so to speak. (Participant 18)

### Theme 5. Recommendations for the development of supportive workplace environments for autistic individuals

Participants offered recommendations to increase the potential for autistic employees to attain a sense of mastery and satisfaction in the workplace.

#### Need for adaptive resources/support

Several participants felt that workplace support could be adaptive, and they highlighted the need for support. This included a responsive environment in which work tasks, social encounters and experiences are malleable and supported, as needed:

The relationships between the people within the workplace, which is where the soft skills support is important and of course . . . the tech work that you’re doing is evolving and changing, so that your needs of tech support will change . . . But it’s not just one or the other, it’s both, that’s sort of going on. (Participant 28)

#### Need for feedback

Workplace support included the need for clear and specific feedback such as detailed guidance to help autistic individuals understand the implications of their actions and engagement. They also described the need for support in being interdependent (e.g. encouraging self-reliance, but being available when needed), yet this support was viewed as needed in a relational and solution-focused manner. Participants highlighted the importance of others in the workplace developing rapport with them (and vice versa) to better ensure that support is commensurate with individual needs, as highlighted by the following participant:

When we first met our support staff, they didn’t really know us, we didn’t really know them so we had to understand each other [through] how they would explain stuff to us, how we’d need to explain stuff to them, and just get along . . . (Participant 13)

#### Need for team-oriented support

Some participants highlighted the importance of support that was offered in a team-oriented way. Given changing workplace personnel, participants emphasised that support should be offered by a variety of personnel. They recounted that support often started a one-to-one approach with a support worker or manager. But as participants adapted to the workplace, they learned to more naturalistically rely on others to help them navigate technical and social tasks. Although managers were viewed as helpful with technical support, several noted that managers also can be supportive in enhancing soft skills within the workplace.

## Discussion

**Figure 1.**
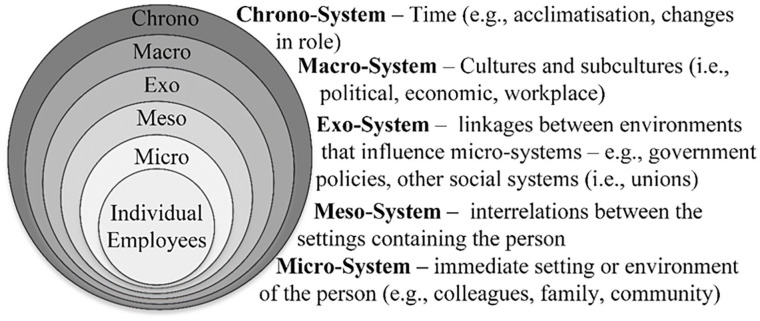
An ecological approach to the workplace experience.

We investigated autistic employees’ experiences in their roles as public-sector employees within two industry-led autism employment programmes. We described their emerging identity as a ‘worker’ or ‘employee’, finding that the interaction between individual and environmental factors shaped employees’ experience of work and their sense of identity as an employee. The dynamic interaction between the individual and their workplace environment is well captured and conceptualised by ecological systems theory (EST; Figure 1; Bone, 2015). Importantly, by referring to EST we can describe the collective, integrative role of stakeholders and systems in contributing to a well-functioning inclusive vocational system, and by extension, employees’ experience and success within that system (Bone, 2015; Nicholas et al., 2018; Yang & Sanborn, 2021). Accordingly, our findings demonstrate how the experience of work and employee identity are reflective of multiple factors, including elements that exist within, and interact across, various ecological levels.

Participants’ experience in the workplace also changed over time. The dynamic nature of the work ecosystem interacted with and influenced workplace experiences, shaping how participants perceived work, engaged within the workplace and with others, as well as how they conceptualised themselves as employees. The experiences of participants were reflective of their individual traits and assets (e.g. age, education level), professional and personal relationships within the workplace, the training and the supports they received, as well as broader factors (e.g. work build, task allocation). Such elements, as they interacted, were seen as evolving and shaping the workplace environment and thus their experience of work. These interactions seemed to shape how autistic employees experienced their job, including job tasks, the social encounters within and outside the workplace and how workplace support was offered, perceived, engaged and experienced. These various elements shaped autistic employees’ sense of job self-efficacy (e.g. confidence, mastery, relationality), sense of integration and ‘settledness’ (i.e. how settled they felt) within the workplace and feelings about their prospective future as an employee within the context of career development. For example, due to higher initial skill sets, some participants in Programme 1 soon found work tasks easy, resulting in unstructured down-time, which they found difficult to manage, leading to feelings of underutilisation. While this varied between sites and participants, procedures within were modified, with some transitioning out of the programme early into competitive employment within the organisation (Hedley et al., 2023).

**Figure 2.**
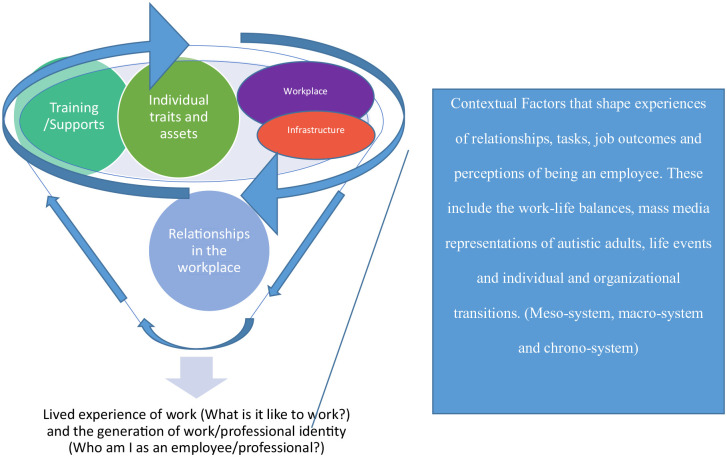
New model of the integrative ecological factors in work experience and the employee identity.

To better capture the current findings, we propose a new model highlighting the integrative ecological factors underpinning autistic people’s work experience and their employee identity (see Figure 2). Employees’ experiences of work and work/professional identity emerged as an integrative journey that included micro-level factors (e.g. learning to effectively complete job tasks and gain independence, relating with others, accessing work supports), meso-level factors (e.g. engagement of various services), macro-system factors (policies and funding for workplace support), exo-system factors (e.g. societal representations of autism, perspectives of autistic people in the context of the workplace) and chrono-system factors (e.g. transitions and life course elements). For instance, autistic individuals’ work productivity at a micro/individual level was shaped by supports and the extent to which they were able to balance work demands with other elements of their social world (e.g. family, school responsibilities, community engagement). Furthermore, how autistic individuals accessed employment support was shaped by the availability of support personnel in the resource structures of society. Identity development processes (e.g. work and autism identity), job self-efficacy and a sense of integration in the workplace developed over time as autistic employees adapted to their work environment yet also were favourably impacted by resource availability, supports and the engagement and understanding of others.

While these findings largely focused on autistic individuals’ experiences and outcomes, results also demonstrate how other individuals within the workplace (e.g. non-autistic co-workers) are also impacted by various ecological factors. For instance, the transience of autism support personnel invariably had an impact on workplace capacity to support and orient co-workers and managers relative to engaging their autistic colleagues. Not having sufficient or ongoing access to autism-focused awareness and professional development likely heightens challenges for other workplace staff to nurture a pro-diversity work environment. Indeed, the sufficiency of autism knowledge and training in the broader workforce was highlighted by some co-workers in Programme 1 as a potential barrier to support the programme (Spoor, Bury, et al., 2021). Such gaps have the potential to diminish outcomes and morale at various levels: individually (e.g. how positive and driven the autistic employee is to excel and grow at work) and collectively (e.g. how prepared and motivated colleagues are to work together inclusively as a diverse and mutually supportive team).

Workplace needs are variable, nuanced and emerge as the workplace context changes. Multiple and evolving influences shaping the work experience suggest that workplace interventions need to be holistic, developmental and supportive of interdependence. These findings demonstrate the value of an ecological approach in understanding and advancing workplace experiences in autism employment programmes, with the proposed model providing what contextual factors may shape employee experiences. Exploring malleable characteristics in the broader system may inform employment support design and capacity-building opportunities, including the interrogation and development of inclusive work. Accordingly, work supports should be expanded in building system resource capacity. The broad-based challenges faced by autistic employees invite expanded capacity-building support to various relevant players such as managers and co-workers, instead of our traditional focus largely on autism-focused support workers in building workplace capacity (Bury, Flower, et al., 2021). Focus effort towards environmental and experiential change can be more readily operationalised by multiple strategies and stakeholders committed to capacity building. Furthermore, the addition of technology solutions (e.g. apps) to provide tailored workplace support may be helpful, but may require refinement to determine what, how and when such workplace support can be offered for optimal benefit to autistic employees or others in the workplace (Gentry et al., 2015; Spoor et al., 2024; Spoor & Walkowiak, 2024).

Implementation of workplace supports must also consider individual and workplace assets. Such assets can support one’s work productivity and sense of belonging in the workplace. For instance, several participants described how prior education (e.g. a university training) or work experience contributed to their successful adaptation to the workplace. Furthermore, co-workers’ a priori knowledge about autism and/or relevant personal experience contributed to greater understanding and proactivity. Similar sentiments were evident from the perspective of co-workers in these same programmes (Spoor, Bury, et al., 2021). Such findings provide support for workplace consideration of individual and environmental assets in designing workplace support (Nicholas et al., 2018; Vogus & Taylor, 2018).

Building a proactive work environment requires autism education/professional development for non-autistic colleagues (Flower et al., 2019). Reflected in these current findings, knowledge-advancement supports broadened autism knowledge and, in some cases, resulted in greater understanding. However, this research suggests such education needs to avoid clinical, behavioural, pejorative, or negative stereotypical portrayals of autism. Attention to education content and process requires empowering and inclusive messages that amplify the contributions and assets of autistic colleagues, as well as diverse perceptions of autistic individuals working across a wide range of work settings.

### Study limitations

First, while a strength of our study was the number and depth of interviews with autistic participants in differing industry-led employment programmes, spread across several different sites, our study was also limited by the fact that we only interviewed autistic participants. To address this limitation, we have attempted to integrate our current findings with other research by our team which has specifically examined the perspectives of non-autistic co-workers. Nonetheless, while our focus on autistic participants provided insight into interacting systems that shaped their experiences, broader data elicitation and focus could amplify other relevant perspectives in the workplace. Further study of other individuals (e.g. managers, co-workers, support workers) and other salient elements (e.g. workplace culture, diverse industry/employment sectors) may amplify the range of individual, relational, environmental and discursive factors that may have a bearing on outcomes, and ways that work could be co-shaped in fostering inclusive workplaces. Second, although several sites and programmes were included in this research, we must acknowledge that our findings are limited by our reliance on data from two programmes operating in similar, white-collar government agencies, thereby affecting generalisability to other settings. In addition, although similar, the programmes differed somewhat in terms of the specific models of support they provided. Replication across broader workplace and regional contexts is therefore warranted.

Third, participants were also generally well educated and male (88%); while this may be reflective of the industry, it represents a potential limitation of the data. Future research should consider expanding this model into similar programmes in other industries, or with autistic employees with co-occurring conditions not represented here (i.e. intellectual disability).

### Future research and conclusions

Future research is needed to illuminate how the environmental/ecological elements described here can enhance workplace understanding and outcomes for autistic employees, including the extent to which ecological factors are similar or different for autistic versus non-autistic employees could be explored as well as the granularity of differences across the autism spectrum. Importantly, we provided a new ecological systems model to enable better understanding of how environmental factors interact with and predict employment outcomes across diverse workplace sectors (retail, trades, professional, hospitality, etc.). Future research should explore adaptative strategies and infrastructures that organisations have implemented to accommodate the workplace needs of autistic individuals in a dynamic work environment. Unpacking the subjective, relational and structural factors that shape individual/environment relationships and experiences is needed. Practically, it is hoped that this focus of inquiry will shed light on ways to improve practice and the integration of supportive resources and policies in the aim of more inclusive workplaces.

## Notes

1. There is significant variability in preferences for describing autism (Bury, Jellett, Haschek, et al., 2023; Bury, Jellett, Spoor, & Hedley, 2023). Consistent with the Publication Manual of the American Psychological Association 7th edition, we use both person-first (e.g. person on the autism spectrum) and identity-first (e.g. autistic person) language.
